# Advancing Health Equity and Policy through Integrative Global Ecological Analysis with Multidisciplinary Databases

**DOI:** 10.34133/research.1089

**Published:** 2026-01-23

**Authors:** Lei-Ming Cao, Yi-Fu Yu, Lin-Lin Bu

**Affiliations:** ^1^ State Key Laboratory of Oral & Maxillofacial Reconstruction and Regeneration, Key Laboratory of Oral Biomedicine Ministry of Education, Hubei Key Laboratory of Stomatology, School & Hospital of Stomatology, Wuhan University, Wuhan, China.; ^2^ Department of Oral & Maxillofacial - Head Neck Oncology, School & Hospital of Stomatology, Wuhan University, Wuhan, China.

## Abstract

Global public databases now contain extensive information about disease, environment, and society, but much of these data have been used only for descriptive summaries rather than for exploring deeper causes of health differences between countries. This commentary discusses how combining data from multiple international sources can reveal new patterns that support fairer and more effective health policies. Using 2 recent examples, we show how this approach can be used to answer important public-health questions, fill gaps in existing databases, and translate scientific evidence into preventive policy. Future ecological studies should continue to connect health data with environmental and social indicators to better understand and reduce global health inequalities.

## Introduction

The growing availability of comprehensive global databases such as the Global Burden of Disease (GBD), the Global Cancer Observatory (GCO), the Food and Agriculture Organization Corporate Statistical Database (FAOSTAT), and the Global Dietary Database has enabled large-scale assessments of health, environment, and development across over 200 countries [1–4]. Yet most studies remain isolated and descriptive, summarizing temporal trends without exploring how environmental, social, or economic contexts shape population health. To advance beyond this “descriptive ceiling”, a new framework that integrates multidisciplinary global datasets and translates findings into actionable public-health policy is needed.

Although cohort studies provide high-quality evidence on individual-level risk, their translation to national or global policy is often indirect. Cohort findings are typically context specific, geographically constrained, and difficult to extrapolate across regions. In addition, many low- and middle-income countries lack the resources to establish and maintain large, long-term cohorts. Ecological studies offer a complementary approach by analyzing exposures and outcomes at the population level. Using countries or territories as the unit of analysis aligns evidence generation with the administrative units responsible for public-health governance, resource allocation, and regulatory action, thereby facilitating more direct policy translation. However, ecological studies are subject to recognized limitations, including ecological fallacy, residual confounding, collinearity, temporal ambiguity, and variable data quality [5]. Nevertheless, the maturation of global databases has begun to mitigate several of these concerns. Standardized data collection improves cross-country comparability, longitudinal coverage enables trend and sensitivity analyses, and integrating multiple databases allows multidimensional assessment of complex population-level determinants that are not observable within single-domain studies [6].

This commentary proposes an integrative global ecological analysis (IGEA) framework that leverages multidisciplinary global databases to examine population-level associations between disease burden and environmental, social, and economic factors. The goal is to move beyond repetitive descriptive analyses and provide interpretable macrolevel evidence to inform and advance public-health policy.

## Lessons from 2 Global Ecological Studies

Cao et al. [7] integrated data from FAOSTAT with the GBD and GCO databases to examine the correlation between national areca nut production and oral cancer incidence. This analysis partly addressed a long-standing gap in the GBD dataset, which lacks information on areca nut consumption, an established carcinogenic factor, and offered quantitative evidence supporting tighter regulation of areca nut production and trade as a public-health measure. This study established a supply-side association between areca nut and the incidence of oral cancer, thereby supporting potential regulatory strategies such as taxation on areca nut production and importation.

Li et al. [8] used population-by-altitude distribution data to develop a new ecological indicator, the population altitude index, representing population-weighted mean residential altitude. By combining this index with GBD data, they demonstrated a positive association between national-level residential altitude and orofacial cleft birth prevalence. This work translated well-established individual-level evidence, that higher-altitude residence increases cleft risk, into population-scale validation. Moreover, it introduced a reusable quantitative framework for global ecological epidemiology, enabling future analyses of altitude-related diseases and other environmental determinants of health. These findings provide evidence to inform strengthened maternal care and screening policies in affected countries and territories.

This type of ecological research generally follows a sequence of 5 steps.

1. Identify a policy-relevant question. A research question is identified by selecting a risk factor or association that has strong individual-level evidence but lacks robust validation at the global or population level. The question must be framed with potential policy implications in mind.
2. Data acquisition and extraction. Corresponding data are obtained from high-quality international databases to extract comparable national or regional indicators of both the exposure and the health outcome. Table 1 outlines several global data sources that provide variables for this approach.
3. Data harmonization and preparation. The multidisciplinary datasets are harmonized to ensure consistency in time frame, geographic boundaries, and variable definition.
4. Ecological analysis and confounding control. Ecological analyses are performed, such as correlation, trend, or multivariable modeling. This stage needs comprehensive sensitivity analysis to evaluate associations and adjust for potential confounding variables.
5. Policy interpretation and translation. The resulting insights are interpreted in a broader context to inform public-health policy and promote health equity, thereby transforming individual-level evidence into globally validated, actionable insights.

**Table 1.** Global data sources that may contribute to ecological research

Database sources	Provider	Link
FAOSTAT	Food and Agriculture Organization of the United Nations	https://www.fao.org/faostat/en/
Global Dietary Database	The Global Nutrition and Policy Consortium	https://globaldietarydatabase.org/
UNdata	United Nations	https://data.un.org/
The World Factbook	Central Intelligence Agency	https://www.cia.gov/the-world-factbook/
TerraClimate	Climatology Lab	https://www.climatologylab.org/terraclimate.html
LandScan Global	Oak Ridge National Laboratory	https://landscan.ornl.gov/
Human Development Data	United Nations Development Programme	https://hdr.undp.org/data-center
The Global Burden of Animal Diseases Database	World Organisation for Animal Health	https://www.gbadske.org/

FAOSTAT, Food and Agriculture Organization Corporate Statistical Database

## Proposed Principles for IGEA

To enhance rigor and practical relevance, we recommend 6 principles: (a) ground hypotheses in prior biological and individual-level evidence, using cross-level comparisons while minimizing ecological fallacy; (b) maintain equity and policy orientation, prioritizing questions that inform public decision-making rather than nonactionable associations; (c) implement comprehensive sensitivity analyses, leveraging both cross-sectional and longitudinal data while addressing confounding and collinearity with appropriate models and transparent interpretation; (d) ensure rigorous data stewardship by using validated databases, critically appraising their methodologies, and carefully managing heterogeneity and evolving country or territory definitions, ideally prioritizing raw values over transformed metrics to preserve fidelity; (e) acknowledge that conclusions may shift with new data releases and update analyses when major revisions occur; and (f) interpret results cautiously, especially when ecological findings diverge from individual-level evidence, by probing plausible sources of discrepancy rather than treating one scale as refutation of the other.

## Conclusion

Through robust use of multidisciplinary global data and a commitment to policy-relevant inquiry, ecological research can make meaningful contributions to health equity and evidence-based public-health decision-making. Future work should focus on developing additional ecological indicators and statistical models suited to predominantly continuous public databases, and on applying IGEAs across a broader range of diseases.
